# Impact of body image on the kinematics of gait initiation

**DOI:** 10.3389/fnhum.2025.1560138

**Published:** 2025-03-17

**Authors:** Kyosuke Oku, Shinsuke Tanaka, Yukiko Nishizaki, Chie Fukada, Noriyuki Kida

**Affiliations:** ^1^Faculty of Arts and Sciences, Kyoto Institute of Technology, Kyoto, Japan; ^2^Institute for Liberal Arts and Sciences, Kyoto University, Kyoto, Japan; ^3^Faculty of Information and Human Sciences, Kyoto Institute of Technology, Kyoto, Japan

**Keywords:** motor image, motor planning, eyes close (EC), walking, step length, foot height

## Abstract

In daily life, we walk naturally by considering our physical characteristics and formulating appropriate motor plans. However, the impact of changes in body image on walking movements during motor planning remains poorly understood. Therefore, in this study, we examined changes in walking behavior under different conditions where body image was altered. We included 26 participants (13 men and 13 women, aged 18.27 ± 0.52) who performed walking movements under five conditions: eyes open, eyes covered, eyes covered while imagining their bodies becoming larger, eyes covered without imagining altered body size, and eyes open again. As a result, under the condition where participants imagined their bodies becoming larger, their step length, step completion time, and foot lift height increased. To generate a torque larger than the actual body size, the participants made a motor planning with a larger body image, resulting in an increase in step length. Since these results are attributed to the disparity between actual body size and body image, which affects motor planning, our findings have potential applications in rehabilitation and sports coaching settings.

## Introduction

Humans perform walking movements without conscious effort, especially when considering their own physical characteristics and intended movements. Research suggests that this motor planning and motor imagery have equivalent functions (Jeannerod, 1994; Steenbergen et al., 2013; Spruijt et al., 2015). Decety et al. (1994) indicated that mentally imagining a movement activates the same brain regions as when actually performing it (Decety et al., 1994). Movement imagery is defined as “the mental expression of movement without actual bodily movement” (Guillot and Collet, 2005). Considering one’s physical characteristics is considered important in motor imagery.

Walking movements are planned through reverse calculation from the desired speed. These movements are goal-oriented, involving the lower limb. Step length and frequency are adjusted to align with the walking goal at a specific speed (Bruderlin and Calvert, 1989). Previous studies have particularly focused on the transition from a stationary state to the first step taken (Breniere and Do, 1991; Mickelborough et al., 2004; Hass et al., 2008; Vrieling et al., 2008; Ceccato et al., 2009; Bayot et al., 2018; Perera et al., 2023). Research into gait initiation has shown that the greater the electromyogram of the calf muscle of the supporting leg, the faster the forward walking speed (Lepers and Breniere, 1995). Furthermore, studies on the center of pressure (CoP) of the body have indicated that the transition of CoP before walking initiation is related to forward walking speed (Lyon and Day, 1997; Caderby et al., 2014; Yiou et al., 2017). Based on these findings, walking is planned based on the speed and executed while simultaneously controlling the center of gravity and the related muscles.

Motor imagery can encompass various types of movements, and walking movements are particularly useful during rehabilitation. Motor imagery is a useful approach for promoting motor function recovery after cerebrovascular disorders, particularly in relation to walking movements (Peters and Page, 2015). Motor imagery training, involving dorsiflexion and internal/external rotation of the hand joints, improves voluntary control of paralyzed limbs in stroke patients (Stevens and Stoykov, 2003). In a study targeting individuals aged 65 years and above, the incorporation of motor imagery into physical training significantly improved balance ability, walking function, and self-efficacy in preventing falls (Nicholson et al., 2019; Oh and Choi, 2021). Thus, motor imagery is considered effective in improving walking movements.

Recognizing one’s own physical characteristics is crucial to motor imagery. This has been investigated using the concept of body image. Body image is defined as the conscious brain representation of the body, including self-image and appearance perception, which is derived from one’s posture perception (Naito et al., 2016). The discrepancy between body image and actual physical condition is assumed to affect motor performance. For example, in the case of walking, a decrease in step length has been observed when stilts are used to extend the length of the lower limbs (Dominici et al., 2009), because the body image remains the same as the original body. However, the actual body has been extended, resulting in a failure to generate the necessary torque. Thus, the discrepancy between body image and actual physical condition may affect motor performance.

To manipulate body image, visual information plays a crucial role. A person’s body image is thought to be changed by the height of the visible perspective and the presence or absence of visual information in the surrounding environment. Blocking visual information also has a significant impact on movement. Walking with closed eyes results in longer step length and longer time per step (Perry et al., 2001; Wuehr et al., 2013). Additionally, Nishizaki et al. (2022) indicated that raising viewpoints through virtual reality (VR) can make individuals perceive themselves as larger than their actual size, causing increased step length (Nishizaki et al., 2022). Similarly, instructing individuals that their body has become large while raising the viewpoint through VR has also been shown to increase their step length (Sasaki et al., 2021). Thus, visual information plays a significant role in walking movements and posture control, and body image can have a significant impact on movement.

This study aimed to investigate the relationship between motor imagery and motor output by comparing the kinematics of initial walking in the open-eye state, closed-eye state, and closed-eye state with manipulated body image. Participants in this study were asked to imagine an enlarged body image with simple verbal instructions only, and the impact on initial walking was examined. To facilitate imagining an enlarged body image, we blocked visual information to avoid surrounding influences on walking movements (Prokop et al., 1997; Mohler et al., 2007) and achieve more discernible changes. By investigating the kinematics of movements in a state where visual information is blocked and body image is changed, the aim was to elucidate the mechanism of motor output. It was hypothesized that if the body image is larger than the actual body size, the step length and height of lifting the foot would increase. Clarifying the mechanism of motor output is expected to have applications in rehabilitation and sports.

## Materials and methods

### Participants

The experiment involved 26 healthy adults (13 men and 13 women, aged 18.27 ± 0.52 years). Participants were allowed to use either their dominant or non-dominant foot for the first step. The sample size was determined based on previous studies (*n* = 26) (Rochat, 1995; Gabbard and Ammar, 2005; Gabbard et al., 2005; Leclere et al., 2019, 2021). The experiment was conducted between October 7, 2023, and October 16, 2023. Prior to the experiment, the purpose and procedure were explained to the participants, and their written consent was obtained. The study was approved by the Ethics Committee of Kyoto Institute of Technology and was conducted in accordance with the Declaration of Helsinki.

### Experimental setup and procedure

**Figure 1:**
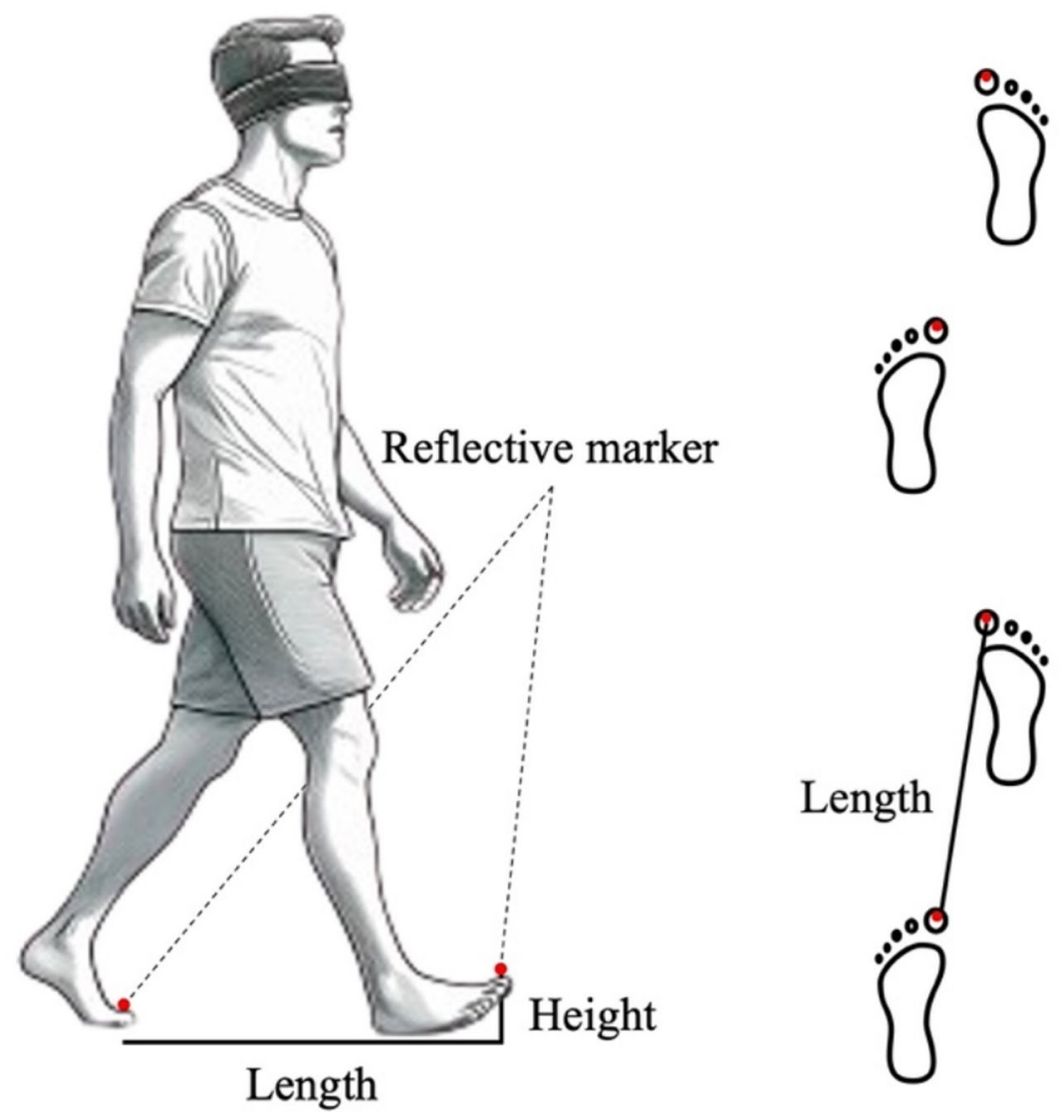
Overview of the experimental setup. Reflective markers were attached to both toes of the participants, and under the eyes-covered state, they were asked to walk while wearing a blindfold.

A schematic of the experiment is presented in Figure 1. The starting point was marked with tape, and the walking tasks were performed barefoot. There were two reasons for conducting the experiment barefoot: firstly, to avoid getting feedback on the weight of the shoes, and secondly, to be able to attach the infrared markers directly to the feet when measuring the kinematics. The tasks started in an upright position with both toes aligned, followed by four steps, then stopped with both feet aligned. The speed was set at a natural walking pace. The leading foot was allowed to step out naturally, without considering the dominant foot. There was also no bias observed in terms of whether the right or left foot was used to start walking, regardless of which foot was dominant. Two infrared cameras (OptiTrack V120 DUO, NeuralPoint) were placed approximately 6 m in front of the participants to capture the walking movements. Reflective markers with a diameter of 16 mm were attached to the tips of the second toes of both feet to obtain three-dimensional (3D) coordinates. These markers were tracked by the two infrared cameras, and the data were obtained with a time resolution of 120 Hz (Gonno et al., 2022).

The experiment consisted of five phases and the conditions of each are summarized in Table 1. In Phase 1, participants were asked to perform 10 walking tasks in which they took four steps with their eyes open. In Phase 2, they were asked to wear a blindfold and perform 10 walking tasks with their eyes covered. After the trial, they opened their eyes and returned to the starting point on their own. Next, in Phase 3, while still wearing the blindfold and maintaining the same body proportion, participants were instructed to walk 10 times while imagining their bodies becoming larger, as if their heads would touch the ceiling of the laboratory (approximately 4 m in height). In order to help participants imagine themselves as larger, we gave them 10 s to imagine themselves each time they tried. Subsequently, in Phase 4, they were asked to perform three walking tasks using their original body image, while still wearing the blindfold. Finally, in Phase 5, participants were asked again to perform three walking tasks without the blindfold and again using their original body image. This sequence was fixed to investigate whether there was a lasting change in behavior, and Phases 4 and 5 were carried out to rebuild participants’ body image. After the experiment, participants completed a short self-report survey to assess their ability to imagine their bodies getting larger. They rated their level of success on a 5-point Likert scale (1 = not at all, 5 = very well). They were also asked to provide open-ended comments about their subjective experience of imagining an enlarged body.

**Table 1:** Presence or absence of blindfold and bigger body image in each phase.

	Phase 1	Phase 2	Phase 3	Phase 4	Phase 5
Blindfold	−	+	+	+	−
Bigger body image	−	−	+	−	−

+ Presence, − Absence.

### Data analysis

The post-experiment survey consisted of a 5-point Likert scale (1 = not at all, 5 = very well) assessing participants’ ability to imagine their bodies as larger. Out of 26 participants, 20 reported a score of 3 or higher, indicating moderate to successful imagining of an enlarged body. Therefore, only the data from these 20 participants were included in the subsequent kinematic analysis. We focused only on the first-step movement in each walking task owing to the possibility of body image impairment by somatosensory feedback during walking. The time-series 3D position data of the infrared markers on the second toes were preprocessed using a second-order Butterworth low-pass filter with a cutoff frequency of 10 Hz (Yamamoto and Kushiro, 2014). The start of the movement was defined as the onset, when the speed consistently exceeded 30 cm/s for the first 5 frames; the end of the movement was defined as the offset, when the speed consistently remained below 30 cm/s for 5 frames. This threshold of 30 cm/s was based on our previous research into lower limb movement (Oku et al., 2023a,b). The following indices were calculated for each trial: step length (Length)—distance between the markers attached to both toes at the end of the first step; maximum height of the toes (Maximum Height)—maximum height from the ground during the swing phase from the onset to offset of the first step; time taken for one step (Time)—time from the start to the end of the first step. The average values were calculated for each condition.

### Statistical analysis

To assess the reliability of the measurements, we calculated both the coefficient of variation (CV) and the intraclass correlation coefficient (ICC(1,k)). The CV was used to examine whether the within-participant variability of the movements changed depending on the phase. The within-participant CV for each variable (step length, maximum height, step time) was calculated for each condition (Phases 1–5) and averaged across participants. A one-way analysis of variance was then performed on the CV of each variable, with phase as a factor. When the main effect was confirmed for each variable, Bonferroni multiple comparisons were performed across phases. However, it is not possible to assess the reliability of performance within a phase based on the CV alone. Therefore, we also calculated the ICC(1,k) to examine the reliability of the 10 repetitions of performance per participant in each phase.

In addition, to examine changes in gait as a function of phase, we performed a one-way analysis of variance with phase as a factor for step length, time and maximum height of the first step. As step length, time and maximum height are each independent variables, we used a one-way ANOVA rather than a MANOVA. When the main effect was confirmed for each variable, Bonferroni’s *post hoc* comparison was performed across phases.

## Results

**Figure 2:**
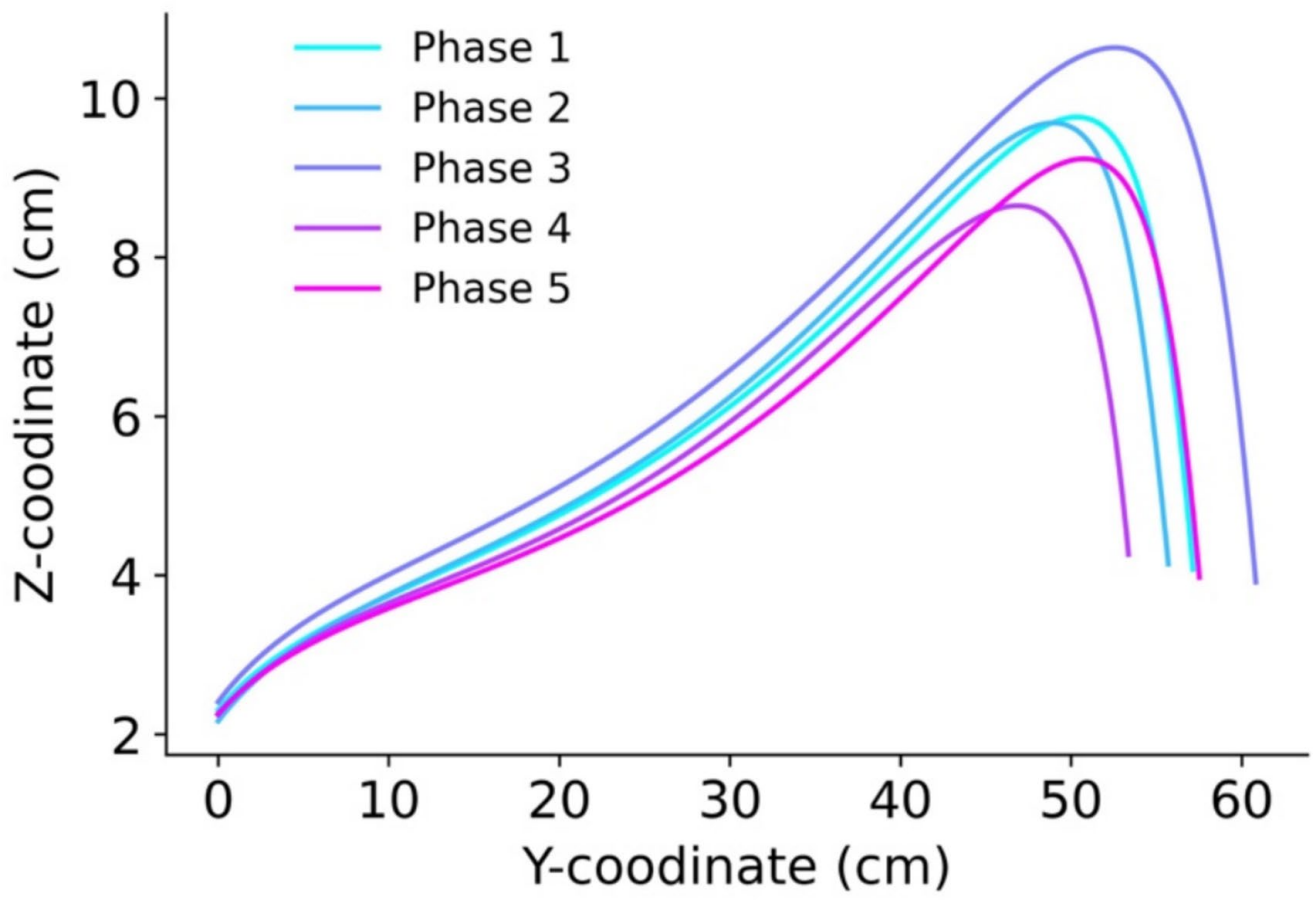
The trajectory of motion in the sagittal plane (Y-Z direction). The line colors represent the phases: *Phase 1* (eyes open), *Phase 2* (eyes covered), *Phase 3* (enlarged body image with eyes covered), *Phase 4* (eyes covered post Test 1), and *Phase 5* (eyes open post Test 2).

The motion trajectory in the sagittal plane (Y-Z) is shown in Figure 2. When participants assumed a large body image, the step length increased along with the possibility of higher foot elevation. Additionally, examination of the motion trajectory revealed that instead of following a parabolic curve, the foot can be brought up to the landing point and then lowered straight down.

**Figure 3:**
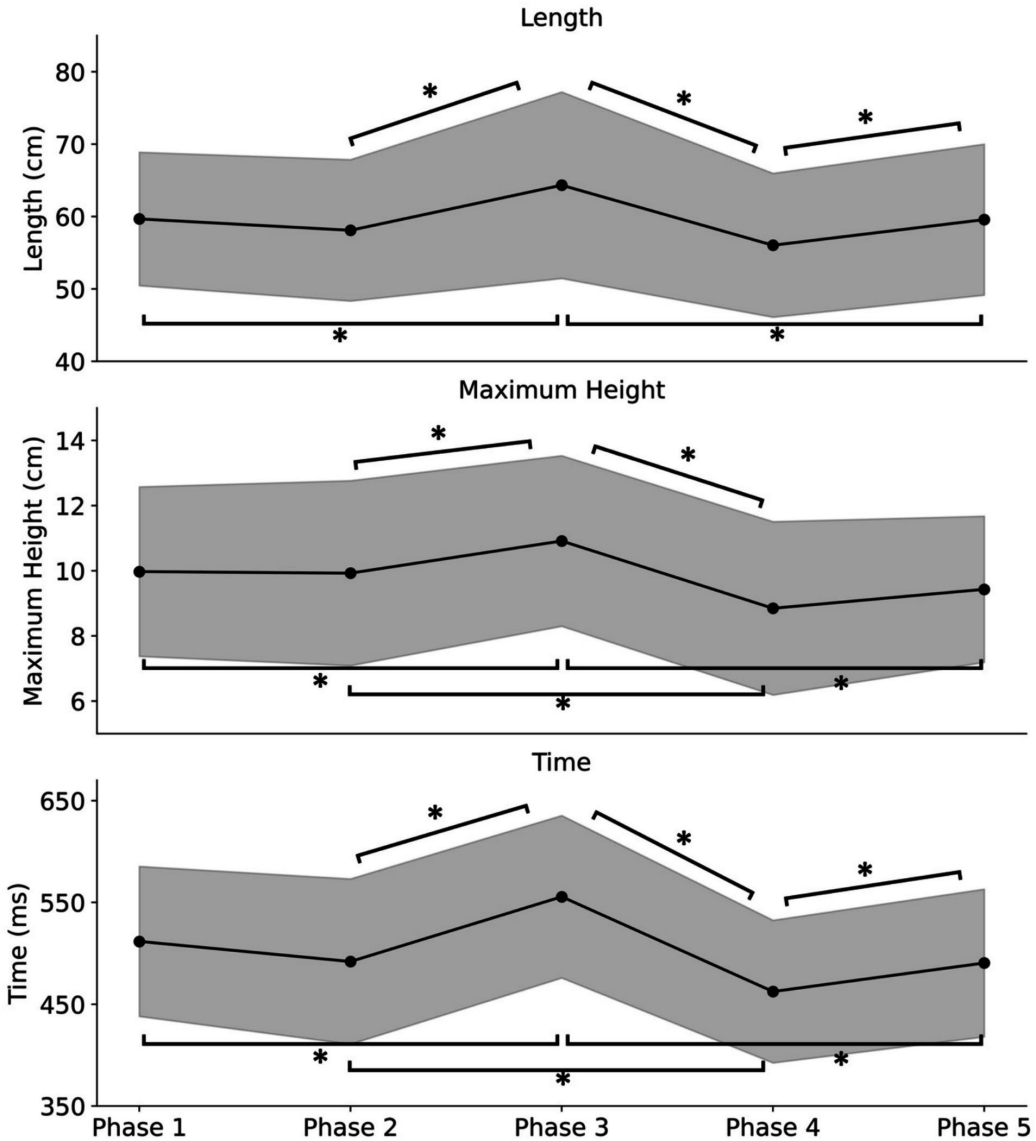
The average values of step length (Length), foot lift maximum height (Maximum Height), and time per step (Time) among participants for each phase. *Phase 1* (eyes open), *Phase 2* (eyes covered), *Phase 3* (enlarged body image with eyes covered), *Phase 4* (eyes covered post Test 1), and *Phase 5* (eyes open post Test 2). The bands represent the standard deviation. Single asterisks (*) represent *p* < 0.05.

Figure 3 shows the average step length across participants for each phase. A one-way ANOVA was conducted with phase conditions as factors to determine how step length varied across conditions. The analysis revealed a significant main effect [*F* (4, 76) = 13.68, *p* = 1.90 × 10^−8^, *η^2^* = 0.07]. Subsequently, Bonferroni’s multiple comparisons test revealed significant differences between Phases 1 and 3, Phases 2 and 3, Phases 3 and 4, Phases 3 and 5, and Phases 4 and 5 (*p* < 0.05). In addition, no significant difference was found between Phases 1 and 5 (*p* > 0.05).

The average height at which the participants lifted their feet for each condition is also shown in Figure 3. The one-way ANOVA with the phase conditions as factors revealed a significant main effect [*F* (4, 76) = 11.13, *p* = 3.70 × 10^−7^, *η^2^* = 0.07]. Subsequently, Bonferroni’s multiple comparisons test revealed significant differences between Phases 1 and 3, Phases 2 and 3, Phases 2 and 4, Phases 3 and 4, and Phases 3 and 5 (*p* < 0.05). In addition, no significant difference was found between Phases 1 and 5 (*p* > 0.05).

The results for average time required for the first step in each phase is also presented in Figure 3. The one-way ANOVA with the phase conditions as factors revealed a significant main effect [*F* (4, 76) = 26.54, *p* = 8.67 × 10^−14^, *η^2^* = 0.15]. Subsequently, Bonferroni’s multiple comparisons revealed significant differences between Phases 1 and 3, Phases 2 and 3, Phases 2 and 4, Phases 3 and 4, Phases 3 and 5, and Phases 4 and 5 (*p* < 0.05). In addition, no significant difference was found between Phases 1 and 5 (*p* > 0.05).

The calculated CV and ICC(1,k) of the participants are summarized in Table 2. CV was calculated to assess measurement variability between conditions, and ICC was calculated to examine measurement reliability. The average CV among participants was sufficiently low for all variables (Length, Height, and Time) under all conditions. Regarding CV, one-way ANOVA was conducted for each variable, and significant main effects were observed for step length [*F* (4, 76) = 4.35, *p* = 0.0032, *η^2^* = 0.113]. The Bonferroni post-hoc comparisons showed that the CV for Phase 4 was significantly higher than that of Phase 5 (*p* < 0.05). For Maximum Height, a significant main effect was observed [*F* (4, 76) = 3.63, *p* = 0.0094, *η^2^* = 0.0974], with Bonferroni *post-hoc* comparisons showing a significantly higher CV for Phase 4 than that of Phase 5 (*p* < 0.05). For the CV of Time, a significant main effect was observed [*F* (4, 760) = 4.74, *p* = 0.0018, *η^2^* = 0.128], with Bonferroni post-hoc comparisons showing significantly higher CV for Phases 2, 3, and 4 than that of Phase 5 (*p* < 0.05). Furthermore, significant correlations were found for ICC(1,k) of each variable during all phases.

**Table 2:** Mean coefficient of variation (mean CV) and intra-class correlation coefficient (ICC(1,k)) of participants for each phase.

Variable	Mean variable	Phase 1	Phase 2	Phase 3	Phase 4	Phase 5
Length	Mean CV (SD)	5.83 (2.19)	5.44 (2.13)	5.23 (2.57)	7.28 (4.39)	4.25 (2.17)
	ICC(1,k) [95% CI]	0.99 ^***^ [0.97, 0.99]	0.99 ^***^ [0.98, 1.0]	0.99 ^***^ [0.99, 1.0]	0.93 ^***^ [0.83, 0.97]	0.98 ^***^ [0.95, 0.99]
Height	Mean CV (SD)	10.44 (4.33)	13.05 (4.86)	11.67 (5.65)	13.14 (6.55)	9.00 (4.56)
	ICC(1,k) [95% CI]	0.98 ^***^ [0.96, 0.99]	0.98 ^***^ [0.95, 0.99]	0.96 ^***^ [0.93, 0.99]	0.92 ^***^ [0.82, 0.97]	0.94 ^***^ [0.86, 0.98]
Time	Mean CV (SD)	6.52 (3.25)	8.78 (4.02)	8.51 (3.64)	9.43 (6.80)	5.09 (2.66)
	ICC(1,k) [95% CI]	0.98 ^***^ [0.95, 0.99]	0.97 ^***^ [0.94, 0.99]	0.96 ^***^ [0.93, 0.98]	0.87 ^***^ [0.69, 0.95]	0.96 ^***^ [0.9, 0.98]

^***^ *p* < 0.001. *Phase 1* (eyes open), *Phase 2* (eyes covered), *Phase 3* (enlarged body image with eyes covered), *Phase 4* (eyes covered post Test 1), and *Phase 5* (eyes open post Test 2).

## Discussion

The result of this study shows that when participants with a body image larger than their actual proportions started to walk, the step length, time taken, and height at which the foot was lifted increased. Furthermore, confirmation of sufficiently low CV and within-condition ICC(1,k) suggests that the participants were able to consistently and effectively imagine a larger body image. In contrast to the findings of the previous study (Dominici et al., 2009), where the step length decreased when using stilts while maintaining the same body image, in this study, the opposite phenomenon was observed. Previous studies have pointed out that body image and motor planning are equivalent functions (Jeannerod, 1994; Steenbergen et al., 2013; Spruijt et al., 2015). To generate a torque larger than the actual body size, the participants made a motor plan with a larger body image, resulting in an increase in step length, which also suggested an increase in leg-lift height.

Despite differences, the concept of “body image” in this study shares some similarities with that of “body schema.” Body image is defined as a conscious internal representation of the body (Naito et al., 2016), and body schema is defined as a pre-conscious internal representation of the body (Head and Holmes, 1911). Body schemas can be changed by various factors. The measurements sometimes make use of the peripersonal space (PPS), which is generated by the body neurons that react to nearby objects (Graziano et al., 1994) and is considered to play a role in self-protection (Graziano et al., 2004). Stone et al. (2018) argued that the reaction speed increases when objects approach the PPS, defining PPS as the space in which reaction speed increases (Stone et al., 2018). Several studies have reported that PPS can expand body schemas when we actively use tools (e.g., a stick) (Iriki et al., 1996; Bonifazi et al., 2007), which suggests that the change in body schema occurs unconsciously. However, since the participants in this study were asked to consciously perform tasks with a larger body image, body schema is not considered to have affected the results.

The results of this study were not what would be expected from previous research on walking behavior during visual occlusion. Previous research has shown that occlusion of visual information has a significant effect on walking behavior. There are reports that occlusion of visual information increases the disruption of COP during postural control while walking (Duarte and Zatsiorsky, 2002; Schmid et al., 2007). Furthermore, when visual information is blocked during walking, the end time of a step increases and the step length increases to stabilize the center of gravity (Perry et al., 2001; Wuehr et al., 2013). Thus, although different from gait initiation, when visual information is blocked, the center of gravity of the body becomes unstable and step length increases in relation to gait and postural control. Previous research on gait initiation has also reported that the maximum speed of the vertical CoM tends to be slower when the eyes are closed (Chastan et al., 2010). Based on this previous research on walking and gait initiation, it was predicted that step length would increase when the eyes were covered, but as significance was not confirmed in Phases 1 and 2 of the present study, it cannot be said that step length changed when the eyes were covered. A possible reason for this is that the walking movements in this study were performed barefoot. Walking barefoot increases the proprioceptive sensation of the soles of the feet, which stabilizes the COP (Franklin et al., 2015). Therefore, it is assumed that the instability of the COP in the eyes-covered condition reported in previous studies did not occur in this study, and that the step length and time did not change between the open and covered eye conditions.

It was also observed that it was difficult to reconstruct the usual body image when visual information was blocked. As mentioned above, there was no significant difference between Phase 1 (eyes open) and Phase 2 (eyes covered), but on the other hand, step length and time were shorter in Phase 4 (eyes covered) than in Phase 5 (eyes open). In addition, walking time was significantly shorter in the second eye covering (Phase 4) than in the first eye covering (Phase 2), and no significant difference was observed in any of the variables between the first eye opening (Phase 1) and the second eye opening (Phase 5). From these results it appears that step length and time decreased significantly in Phase 4, rather than step length increasing in Phase 5. In the results for CV, all variables were significantly greater in Phase 4 than in Phase 5 and there was a large amount of variation between trials. This suggests that when the body image, which had been enlarged in Phase 3, was returned to its original size, the difficulty of reconstructing the body image had an effect and the variation between trials increased. It is thought that it was more difficult to be aware of their normal body image than the body image they imagined in this study, which was so large that their head would touch the ceiling, in the absence of visual information. Thus, it is likely that the difficulty in reconstructing a normal body image without visual information led to a change in movement and more caution, resulting in shorter step lengths and times. The mechanism of walking movements in reconstructing a normal body image in the absence of visual information is a topic for future study.

### Limitations and future prospects

A limitation of this study is that the instructions on body image were provided verbally. This could have caused variations in perception of the body “becoming larger,” depending on the individual. This study mainly targeted kinesthetic motor imagery, but it was not possible to fully control visual motor imagery. In this study, visual information was blocked by blindfolds, but it is possible to reproduce movement in the mind without visual input. Such visual motor imagery includes both first and third person perspectives, but it is thought that there was variation between individuals in terms of which motor imagery was used. The use of VR as a means of controlling such visual motor imagery is a topic for future research. By using VR to visually control the ‘state of having a larger body’ and examining motor output, it will be possible to investigate the effects of different types of motor imagery.

## Conclusion

The aim of this study was to elucidate the mechanism of motor output by investigating the kinematics of movement when visual information is blocked and body image is changed. In this study, we observed that, as the body image became larger, the step length increased, resulting in an increase in time and leg-lift height. These findings suggests that changing body image could have a considerable impact on actual walking movement. This underscores the importance of body image in motor planning and highlights potential applications in rehabilitation and performance optimization, where cognitive strategies might be used to influence physical movement.

## Data Availability

The datasets presented in this study can be found in online repositories. The names of the repository/repositories and accession number(s) can be found at: https://doi.org/10.6084/m9.figshare.24520528.v1.
